# On the particular vulnerability of face recognition to aging: a review of three hypotheses

**DOI:** 10.3389/fpsyg.2015.01139

**Published:** 2015-08-21

**Authors:** Isabelle Boutet, Vanessa Taler, Charles A. Collin

**Affiliations:** ^1^School of Psychology, University of Ottawa, Ottawa, ON, Canada; ^2^School of Psychology, Bruyère Research Institute, Ottawa ON, Canada

**Keywords:** face recognition, aging, contrast sensitivity, familiarity, context recollection

## Abstract

Age-related face recognition deficits are characterized by high false alarms to unfamiliar faces, are not as pronounced for other complex stimuli, and are only partially related to general age-related impairments in cognition. This paper reviews some of the underlying processes likely to be implicated in theses deficits by focusing on areas where contradictions abound as a means to highlight avenues for future research. Research pertaining to the three following hypotheses is presented: (i) perceptual deterioration, (ii) encoding of configural information, and (iii) difficulties in recollecting contextual information. The evidence surveyed provides support for the idea that all three factors are likely to contribute, under certain conditions, to the deficits in face recognition seen in older adults. We discuss how these different factors might interact in the context of a generic framework of the different stages implicated in face recognition. Several suggestions for future investigations are outlined.

## Introduction

**FIGURE 1:**
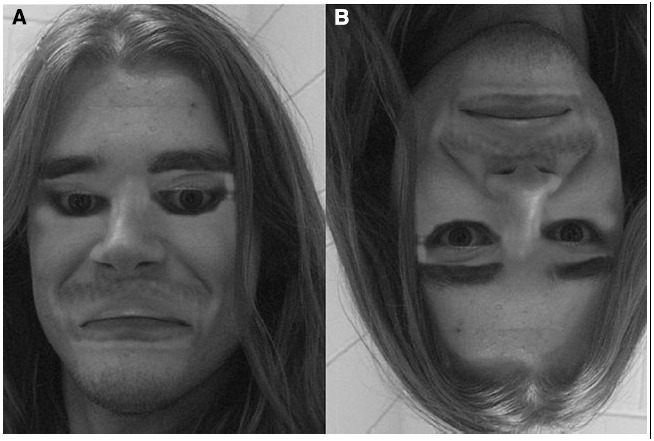
**An example of bizarre experience elicited by faces whose spatial elements are denaturalized. (A)** In the so-called Thatcher Illusion, the eyes and mouth of the upright face are turned upside-down. **(B)** The feeling of bizarreness produced by the manipulation disappears when the image is inverted.

If you ask a layperson whether faces are special, most would not hesitate to answer “*yes*.” Indeed, one does not have to be well versed in the intricacies of visual information processing to appreciate that faces carry a wealth of information relevant for social interactions: information about the emotional status of others, the locus of attention (i.e., via gaze direction), gender, ethnic identity, age, etc. But for most people, the specialness of faces is experienced in reference to the crucial role they play in defining an individual’s identity. Indeed, the keen sense of identity derived from faces is well illustrated by striking examples of individuals who have had to acquaint themselves with a new identity following gross injuries to the face (e.g., Furr et al., 2007), by the bizarre experience elicited by faces whose spatial elements are denaturalized (e.g., Thompson, 1980; Figure 1), as well as by the profound impact that prosopagnosia, the inability to recognize faces, has on those affected as well as their relatives and friends (Yardley et al., 2008).

In the fields of visual perception and cognition, the question of whether there are unique or special visual mechanisms for processing the identity of a face is the topic of considerable scientific debate^1^ (e.g., Damasio et al., 1982; Diamond and Carey, 1986; Gauthier et al., 1999; McKone and Robbins, 2011). Despite ongoing controversy, the layperson’s intuition that faces are special is supported by empirical observations (reviewed by Haxby et al., 2002; Maurer et al., 2002; McKone and Robbins, 2011). First, faces are unique in the sense that they are the only homogeneous stimulus category for which most humans have developed expertise in distinguishing individual members at the subordinate level on a daily basis. Second, faces are unique because their recognition is more severely affected by certain manipulations, a finding that has been attributed to a specialized processing style tailored to the idiosyncratic properties of faces. Finally, faces are unique in that a network of brain areas preferentially activated by faces has been identified.

Multiple studies have reported that older adults have difficulty recognizing faces using a variety of experimental paradigms and stimulus formats. Experimental paradigms have included delayed matching-to-sample with various inspection times (e.g., Smith and Winograd, 1978; Grady et al., 1994; Boutet and Faubert, 2006; Habak et al., 2008; Hildebrandt et al., 2010, 2011, 2013; Obermeyer et al., 2012; Konar et al., 2013), delayed non-matching to sample (Crook and Larrabee, 1992), simultaneous and sequential matching (e.g., Owsley et al., 1981; Chaby et al., 2011), yes/no recognition tests (e.g., Bartlett and Fulton, 1991; Searcy et al., 1999; Boutet and Faubert, 2006; Edmonds et al., 2012; Hildebrandt et al., 2013), as well as naming tasks (e.g., Maylor and Valentine, 1992; Lott et al., 2005). Stimulus formats have included line-drawn faces (Bartlett et al., 1989, 1991), face pictures (e.g., Boutet and Faubert, 2006; Chaby et al., 2011; Edmonds et al., 2012; Obermeyer et al., 2012; Konar et al., 2013), and so-called Mooney faces where color and grayscale information is dichotomized into white or black pixels (Carbon et al., 2013). Age-associated recognition deficits have been reported for test faces presented in a same (all studies presented above) as well as different views (Habak et al., 2008; Meinhardt-Injac et al., 2014).

**FIGURE 2:**
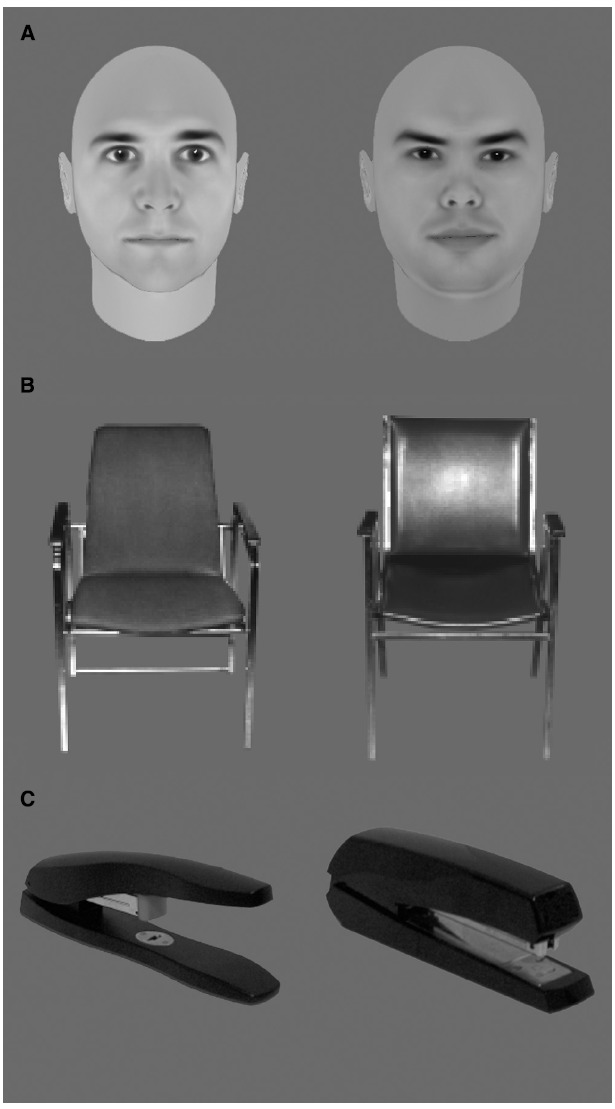
**Examples of stimuli used to trigger within-class discriminations that are equivalent for faces (A) and other complex objects (B,C)**.

Three features of these face recognition deficits are particularly noteworthy. First, studies that have employed yes/no recognition paradigms indicate that this age-related deficit arises primarily from older adults having difficulty rejecting unfamiliar faces, with their ability to correctly recognize familiar faces being comparable to that of younger adults (for a review, see by Searcy et al., 1999). Second, age differences are more pronounced for faces than for other comparable recognition tasks including individual recognition of other objects (chairs and houses: Boutet and Faubert, 2006; watches: Meinhardt-Injac et al., 2014; see Figure 2, for examples) as well as recognition of inverted faces (Grady et al., 1994; Boutet and Faubert, 2006; Chaby et al., 2011, Experiment I; but see Obermeyer et al., 2012). The finding for inverted faces is particularly relevant to our discussion because inverted faces contain the same low-level information as upright ones. Indeed, finding larger differences between younger and older participants for upright relative to inverted faces suggests the implication of higher-order processes involved in normal face recognition *per se*, rather than information demands or lower-level processes. Third, general impairments in cognitive function and object recognition do not completely account for age-related face recognition deficits (Hildebrandt et al., 2011), suggesting that in addition to general functioning impairments (in memory, for example), face-specific factors must also be implicated. The latter two sets of results suggest that faces may also be special in the sense that their recognition appears to be more vulnerable to the aging process than that of other object categories.

The notion that older adults may have particular difficulty recognizing faces may not come as a surprise to those who interact with them on a daily basis: alongside word finding difficulties, trouble with face recognition is one of the most commonly reported complaints in this population (e.g., Chaby and Narme, 2009). What is surprising is that despite multiple investigations into possible underlying mechanisms, researchers have yet to provide an account of the deficit that reconciles the extant literature on normal cognitive aging, the unique mechanisms involved in the face processing, and the nature of age-related deficits in face recognition. The variability in the procedures employed to test face recognition deficits, and in the way that aging affects older individuals, further complicates our understanding of this issue. This paper will examine three hypotheses that have been proposed to explain these deficits, as well as relevant evidence and commentary. The presentation is couched within a generic framework of the different processing stages that are likely to be associated with face recognition and our discussion begins with a brief presentation of this framework. We then review evidence that pertains to each of three hypotheses separately and link them to the different stages proposed in the framework. Our intent is not to provide an exhaustive review of the literature on the impact of aging on face processing but to highlight promising explanations as well as define areas where more research is needed. We also endeavor to demonstrate that investigations into the factors that account for age-related face recognition deficits provide a unique opportunity to advance our understanding of both face-specific processes and aging in general.

## Organizing Framework

Several models of face recognition exist (e.g., Bruce and Young, 1986; Burton et al., 1990; Biederman and Kalocsai, 1997; Riesenhuber and Poggio, 1999; Haxby et al., 2002; Ishai et al., 2005; Wallis, 2013) and our goal is not to integrate theses various models but rather to present a generic organizing context for the review. This framework is inspired by the seminal model of Bruce and Young (1986) but also borrows relevant elements from other models. The framework focuses on aspects of recognition that pertain to the identity of the face and the person it belongs to. As such, it bypasses the identification of a face as a face because our focus here is not on object categorization but rather on recognition of individual faces.

The framework begins with the perceptual analysis of the visual information present in a face for which a recognition judgment has to be made. The perceived face will be analyzed using increasingly complex levels of information that will eventually lead to the formation of a representation. This hierarchical process parallels the increasingly complex response properties of cells along the visual pathway. It is beyond the scope of this presentation to speculate on the exact nature of face representations and we will only mention two types of information that are relevant for this review. The first type of information likely to be extracted from a perceived face is low-level spatial information that corresponds to the filtering properties of cells in early visual areas such as the LGN and primary visual cortex. Second, faces will be decoded on the basis of the information necessary to discriminate this highly homogeneous stimulus category at the individual level. Even though there is an ongoing controversy regarding exact nature of this information, there is substantial evidence that the recognition of individual faces relies on the processing of *configural* information (see reviews by Maurer et al., 2002; McKone and Robbins, 2011). According to Maurer et al. (2002), configural information can refer either to the first-order relations that specify the basic configuration shared by all faces, or to second-order relations (i.e., spatial distances) between facial features, such as the separation between the eyes or between the mouth and the nose. Finally, configural information can refer to holistic information, meaning that when a face is processed, it is as a whole or a Gestalt. We will focus on second-order relations and holistic information in this paper because they are the hallmark of the specialized processing style purported to be associated with face recognition. While different terminology exists in the literature, we will use the term configural when referring to both the second-order and holistic information utilized during face recognition. It is important to note that the exact nature of the information used during face recognition is the topic of considerable debate (e.g., Taschereau-Dumouchel et al., 2010; Wallis, 2013; Xu et al., 2014). In particular, the experimental manipulations used to tap into the processing of second-order relations have been heavily criticized for their lack of realism (Taschereau-Dumouchel et al., 2010). Furthermore, alternative explanations have been put forth to account for some of the findings cited as evidence for holistic face encoding (Xu et al., 2014). Despite this controversy, several studies have examined age-associated face recognition deficits using these same experimental manipulations because of their longstanding presence in the literature on face processing. We are adopting the terms configural, second-order relations, and holistic encoding in this paper to respect the original content of the studies we are reviewing.

Once a representation of the perceived face is formed, it is compared to stored representations for evaluation of the degree of resemblance. If there is a match, a feeling of familiarity will arise. Here, the framework posits that familiarity and recollection can be separated (e.g., Bartlett et al., 2009; Edmonds et al., 2012) in the final decision process. Recognition based on feelings of familiarity does not require the retrieval of additional information regarding the person the face belongs to, the context under which the face was previously encountered, etc. In contrast, a feeling of familiarity might lead to a search for, and retrieval of this information, if the face is indeed known (recollection).

In this review, we present three hypotheses that have been put forth to explain age-related deficits in face recognition. The first hypothesis focuses on low-level processes by stipulating that age-related face recognition deficits are attributable to perceptual deterioration. The second hypothesis focuses on mid-level processes by suggesting that older adults have difficulty recognizing faces because of a deficit in encoding configural information. In the context of the framework described above, both perceptual deterioration and impaired configural encoding would result in impoverished representations of perceived faces and lead to confusion when comparing perceived to stored representations. Finally, the last hypothesis focuses on later stages of the processing stream by stating that older adults have difficulty accessing the contextual information needed to correctly decide whether a face has actually been previously encountered or only feels familiar. We selected these three hypotheses because (i) they can account for different characteristics of the face recognition deficits seen in older adults, (ii) they map onto the different stages presented in the proposed framework, and (iii) they are promising both in terms of their plausibility and of their potential to generate further research. A detailed review of the studies conducted to investigate each of these hypotheses follows.

## Perceptual Deterioration

Impairments in basic sensory abilities have been reported in older adults across all modalities and it has been suggested that perceptual deterioration is a major determinant of age-related cognitive impairments (e.g., Sekuler et al., 1980; Lindenberger and Baltes, 1997; Schneider and Pichora-Fuller, 2000). Past research linking perceptual deterioration with face recognition has focused on visual loss (e.g., Dulin et al., 2011 Wallis et al., 2014), reduced visual acuity (e.g., Tejeria et al., 2002), and reduced contrast sensitivity (e.g., Owsley et al., 1981). Studies investigating the impact of visual loss on face recognition suggest that patients with foveal loss (Wallis et al., 2014), severe peripheral loss (Dulin et al., 2011), unstable fixations (Wallis et al., 2014), age-related macular degeneration (Barnes et al., 2011), and central scotomas (Dulin et al., 2011) display poorer face recognition than controls. It should be noted that these studies may have limited implications for the age-related face recognition deficits that are the focus of this review because participants with self-reported pathological conditions were usually excluded. Furthermore, studies on visual loss are limited by the heterogeneous pathological profiles of the participants, making it difficult to reach generalizable conclusions for the non-pathological aging population. Nonetheless, the finding that visual loss negatively impacts face recognition highlights the need for formal screening of pathological conditions when testing older adults on face recognition tasks.

Studies that have examined the relationship between acuity and face processing using regression analysis in older individuals have yielded inconsistent results (reviewed by Barnes et al., 2011). The results of several studies (e.g., Tejeria et al., 2002; West et al., 2002; Lott et al., 2005; Barnes et al., 2011) indicate low to moderate statistically significant correlations between performance on face recognition tasks and visual acuity. However, Barnes et al. (2011) reported that differences in face identification between younger and older adults disappeared after adjusting for acuity. Tejeria et al. (2002) found that the use of a magnification device improved face recognition abilities in patients with age-related macular degeneration, suggesting that acuity is a determining factor. However, whether these results can be generalized to participants with normal vision remains to be determined. On theoretical grounds, we suggest that reduced acuity is unlikely to contribute significantly to face recognition impairments *per se* because acuity measurements assess only the upper limit of the contrast sensitivity function, and face recognition has been shown to primarily rely on a band of middle relative spatial frequencies, which lie in the middle of the contrast sensitivity function at most common face viewing distances (e.g., Costen et al., 1996; Gold et al., 1999; Näsänen, 1999; Boutet et al., 2003; Collin et al., 2006; Keil, 2009).

Others have suggested that reduced contrast sensitivity (Norton et al., 2009) impairs face recognition in older individuals. Several of the studies cited in the previous paragraph also included contrast sensitivity in their regression models and, as was the case for acuity, found low to moderate statistically significant correlations between contrast sensitivity and face recognition (Lott et al., 2005; but see also Tejeria et al., 2002). Barnes et al. (2011) have also found that differences in face identification performance between younger and older adults disappeared after adjusting for contrast sensitivity. Finally, Lott et al. (2005) reported contradictory findings whereby contrast detection and face recognition were not significantly correlated. Furthermore, contrast sensitivity did not explain more variance in face recognition than age and high-contrast acuity alone.

Owsley et al. (1981) provided a compelling demonstration of a link between reduced contrast sensitivity and face recognition deficits in older adults. In their study, contrast thresholds were measured by asking participants to adjust the contrast of pairs of faces until they could discriminate them. Older participants required significantly higher contrasts to perform the task. Additional results indicated that pairs of faces are equally discriminable by older and younger adults when the faces shown to the older adults are doubled in contrast. This study provides convincing evidence that a decline in contrast sensitivity impedes face recognition in older adults. Using a similar adjustment technique as in Owsley et al. (1981), Owsley and Sloane (1987) demonstrated that reduced contrast sensitivity can account for deficits in processing a variety of real world objects, suggesting that the link between contrast sensitivity and recognition deficits may not be unique to faces.

The above-mentioned limitations notwithstanding, the contribution of low-level visual perception differences to age-related face recognition deficits warrants further investigation. For example, it would be interesting to examine whether older adults rely on the same spatial frequency information as younger adults during face processing. Studies conducted with young adults have shown that face recognition depends on a narrow critical band of relative spatial frequencies in the middle range (e.g., Costen et al., 1996; Gold et al., 1999; Näsänen, 1999; Boutet et al., 2003; Collin et al., 2006; Keil, 2009). It is possible that older adults’ reduced contrast sensitivity for this range as well as for the higher range leads them to rely on lower spatial frequencies during face discrimination tasks. This compensatory mechanism could yield impairments in face recognition because the observers would not be making their judgment on the basis of the band most useful for the task at hand. In addition, such reliance on low spatial frequencies would be most pronounced for faces because object recognition is quite robust to variations in spatial frequency content (e.g., Biederman and Kalocsai, 1997; Collin and McMullen, 2005; Collin et al., 2012). Thus, the low spatial frequencies for which contrast sensitivity is relatively intact in older adults would suffice for object recognition but not face recognition. Another avenue of research would be to mimic the loss in contrast sensitivity associated with aging by presenting younger adults with faces that have been filtered in such a way as to reflect the perceptual experience of older adults. Finding that younger adults display similar impairments in face recognition, and similar brain activation, under such impoverished conditions would provide powerful evidence for the hypothesis that spatial vision loss contributes significantly to face recognition deficits in older adults.

In every day life, it is likely that a host of perceptual problems are actually implicated in the common complaint of face recognition deficits in older adults. However, considering the heterogeneity of the functional deficits in vision that arise with aging, it is important that the factors that are more generalizable in this population, such as changes in the contrast sensitivity function, be dissociated from pathological conditions such as macular degeneration. While we have mapped the perceptual deterioration hypothesis to the first step in the face recognition stream of processing, it should be noted that the way in which perceptual deterioration contributes to cognitive deficits in aging, and whether the former causes the latter, remains to be determined (e.g., Schneider and Pichora-Fuller, 2000). Investigations of age-related deficits in face recognition might actually serve as a model to shed light on this issue.

## Impaired Processing of Configural Information

We began this paper by discussing the special role that faces play in social interactions and visual information processing. We also stated that face recognition appears to be particularly vulnerable to the aging process. Indeed, past research suggests that face recognition deficits are only partially related to other more general impairments (Hildebrandt et al., 2011) and are more pronounced for faces than for other complex stimuli even when equivalent identity-related tasks with comparable performance are employed (Grady et al., 1994; Boutet and Faubert, 2006; Chaby et al., 2011, Experiment I; Meinhardt-Injac et al., 2014). Although these findings need to be replicated, the emerging pattern is that of a special vulnerability for face processing in aging, rendering explanations based solely on global aging mechanisms less tenable. As a result, several researchers have proposed explanations tailored to the processes involved in face recognition. Even though the exact nature of the information employed during face recognition remains a topic of considerable debate, there is substantial evidence that the recognition of individual faces relies on a processing style specialized to deal with the idiosyncratic properties of this task (see, reviews by Maurer et al., 2002; McKone and Robbins, 2011; but see also, e.g., Taschereau-Dumouchel et al., 2010; Wallis, 2013; Xu et al., 2014, for alternative views). More specifically, face recognition appears to rely on configural information. In contrast, object recognition appears to rely more heavily on information about distinctive features and first-order relations, even when comparable within-category tasks are used (e.g., Yin, 1969; Tanaka and Farah, 1993; Maurer et al., 2002).

Combining the idea that faces are processed on the basis of configural information with the finding that face recognition appears to be particularly vulnerable to aging has led researchers to test the hypothesis that age-related deficits in face recognition arise from a failure to encode configural information in this population. While a variety of experimental tests have been used to investigate this hypothesis, our review focuses on those tests that best capture the link between configural information processing and face recognition in aging (see Murray et al., 2010; Carbon et al., 2013, for other tests of configural processing in older adults).

The face inversion effect (FIE) provided one of the first suggestions that faces are encoded using a specialized processing style. The FIE refers to the finding that face recognition is more severely affected by inversion than recognition of other complex objects (Yin, 1969). Despite some controversy, the detrimental impact of inversion on face recognition is generally thought to arise from difficulties in encoding configural information in inverted faces (Farah et al., 1998; Rossion, 2009; but see also, e.g., Gauthier and Logothetis, 2000; Sekuler et al., 2004, for alternative views). Several studies have examined the FIE in older adults. First, Boutet and Faubert (2006) failed to find a difference between older and younger adults on the FIE in two separate experiments using two different non-face object categories and two different tasks with different mnemonic demands. Their results were partially replicated by Hildebrandt et al. (2010) with a large sample (*n* = 151) of older adults. They found that inversion impedes recognition of faces equally in younger and older adults.

Whereas these findings suggest that analysis of configural information is intact in older adults, other studies have produced contradictory results. Particularly noteworthy are the behavioral studies by Chaby et al. (2011) and Obermeyer et al. (2012), as well as the event-related potential (ERP) studies by Gao et al. (2009) and Daniel and Bentin (2012). Starting with the behavioral studies, both Chaby et al. (2011) and Obermeyer et al. (2012) reported a reduced effect of inversion on face recognition in older adults as compared to younger adults. However, these results should be interpreted with caution because a floor effect seems to exist in the results of the study by Chaby et al. (2011; Figure 2) and because the otherwise reliable effect of aging on false alarms was not replicated in Obermeyer et al. (2012). Furthermore, visual inspection of the results in the Obermeyer et al. (2012) study suggests that older adults’ recognition was impaired by inversion but that the omnibus analysis they performed failed to detect such an effect. Unfortunately, they do not report the necessary *post hoc* statistical analyses to verify whether or not the FIE was significantly different across the two age groups.

It is important to note that none of these studies, with the exception of Boutet and Faubert (2006), included an equivalent non-face recognition task (see also Meinhardt-Injac et al., 2014). The original demonstration of the FIE included such a condition, and it is the finding that *inversion has a greater impact on faces than on other objects* that allows for the conclusion that face recognition relies on a specialized processing type. The finding that inversion has a greater impact on face recognition in younger adults relative to older adults might be due to: (i) a failure to encode configural information in older adults, or (ii) to difficulties dealing with the more complex and ecologically invalid task of recognizing inverted faces in this population. Unfortunately, it is impossible to choose between these alternative interpretations without the inclusion of a non-face category condition as part of the study design.

Two ERP studies that have included inverted faces in their protocol (Gao et al., 2009; Daniel and Bentin, 2012) are relevant to the present discussion. These studies took advantage of the N170 effect to investigate face processing in older adults. The N170 effect refers to the finding that the N170 ERP is larger in response to faces as compared to other stimuli (see Rossion and Jacques, 2008, for a discussion of different interpretations of the N170 effect). Both studies found that the N170 effect is equivalent in older and younger adults, a finding that has been challenged elsewhere (Rousselet et al., 2010; Bieniek et al., 2013). Both studies also found some differences between older adults and younger adults with regards to the N170 signals elicited by inverted faces. Whether these findings have implications for the configural information hypothesis is debatable, however, because we have no indication that the ERPs recorded were actually related to the behavioral manifestation of the FIE; indeed, no performance data were reported in either study.

Bearing in mind the strengths of the studies by Boutet and Faubert (2006) and Hildebrandt et al. (2010), the former having included a non-face object category to measure the classical FIE, and the latter having a large sample size, together with the limitations inherent in the other studies, the balance of evidence seems to favor the idea that processing of configural information, as measured by the FIE is intact in older adults. However, in interpreting all of these findings, it must be kept in mind that there is an ongoing debate about the exact mechanisms responsible for the FIE (e.g., Farah et al., 1998; Gauthier and Logothetis, 2000; Sekuler et al., 2004; McKone and Yovel, 2009; Rossion, 2009). In part for this reason, other investigators have focused instead on different tests of configural information processing. We turn to these now.

**FIGURE 3:**
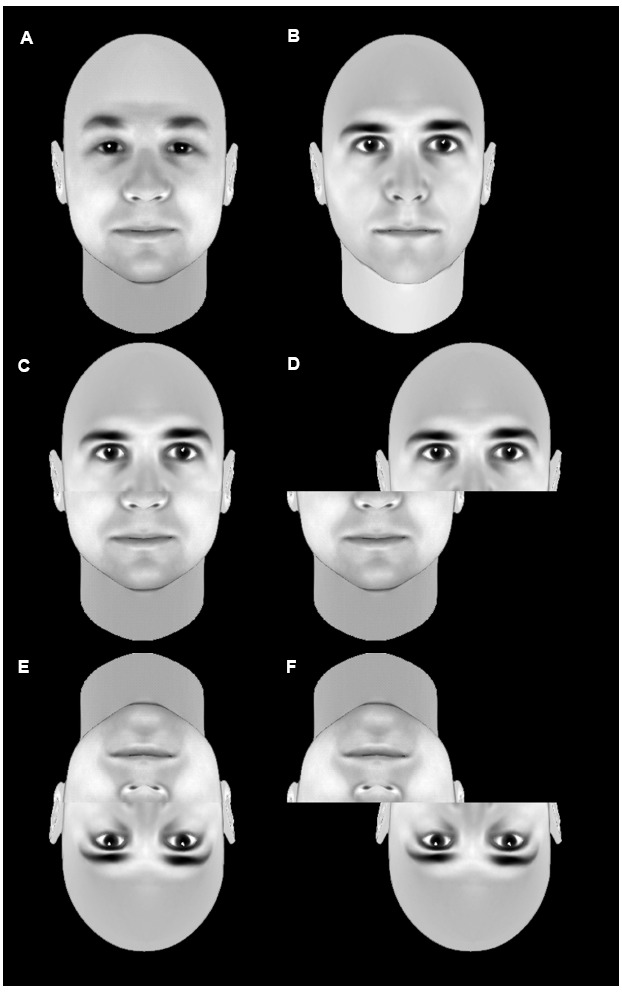
**An example of the composite effect.** The top row shows two unmodified faces **(A,B)**. The middle row shows a stimulus composed of the top half of b and the bottom half of a in an aligned condition **(D)** or a misaligned condition **(D)**. Recognition of the individual faces that make up the composite is significantly less accurate in the aligned composite **(C)** than the misaligned non-composite **(D)**. This difference is less pronounced when the images are inverted **(E,F)**. This is taken as evidence that faces are normally processed holistically, but that inversion disrupts this holistic processing.

The composite effect (Young et al., 1987) and the whole-part advantage (Tanaka and Farah, 1993) have been used to investigate holistic face processing. The composite effect refers to the finding that composites made of two aligned half faces are more difficult to recognize than non-composites made of two misaligned face halves (Figure 3). The whole-part advantage refers to the finding that recognition of facial features is easier when they are presented in full faces rather than in isolation. Both these differences are reduced when faces are inverted, suggesting that recognition of upright faces is performed on the basis of a unitary holistic representation and that formation of this representation is impaired by inversion.

Inconsistent patterns of results have arisen from studies that have employed these tests to investigate holistic encoding in older adults. First, Konar et al. (2013) and Wiese et al. (2013) have provided evidence that the composite effect is present in older adults. In contrast, Boutet and Faubert (2006) and Hildebrandt et al. (2010) failed to find a significant composite effect in older adults, though a trend in the direction of a composite effect was found in the former study. Contradictions also exist for the whole-part advantage: whereas, Boutet and Faubert (2006) found that the whole-part advantage is equivalent in older and younger participants, Hildebrandt et al. (2010) found no difference between recognition of parts in isolated vs. whole conditions in older adults. It thus appears that the literature to date provides inconclusive evidence with respect to holistic encoding of faces in older adults. Finally, to our knowledge, only one study has examined sensitivity to second-order relations in older adults. Specifically, Hildebrandt et al. (2010) measured older adults’ sensitivity to changes in the spatial relation between facial features. Their results indicate that older and younger adults are equally sensitive to changes in second-order information.

It is difficult to reconcile these findings as they relate to the hypothesis that face recognition deficits in older adults arise from difficulties with processing configural information. Nonetheless, a number of general conclusions and suggestions can be made. The main pattern that arises from our review of the literature is that the same tests of configural information often yield inconsistent results in older adults. These discrepancies can arise either from the status of the sample of participants tested or from the test parameters employed. Starting with the status of the sample, it is now becoming increasingly obvious that substantial heterogeneity exists within “normal” aging (e.g., Ardila, 2007; Nyberg et al., 2012). To further complicate matters, the variability in older adults might arise from the fact that some of the adults tested may actually be on a trajectory of pathological aging. Indeed, the Mini-Mental State Exam, which is widely used to screen out such pathologies from the studies reported here, has poor sensitivity to the early stages of Alzheimer’s disease and mild cognitive impairment (e.g., de Jager et al., 2009). As discussed in the previous section, old age also comes with a variety of vision problems that the participants may not be aware of but that can nevertheless have an impact on face recognition.

With respect to the test parameters employed, our review of the literature reveals a variety of stimulus manipulations and testing conditions that make it difficult to determine whether two different studies actually tap into the same processes. For example, the composite effect seems to be highly sensitive to specific stimulus and methodological parameters, and several modifications of the original paradigm (Young et al., 1987) have been published. The effect can be tested using a naming task, a short-term recognition task, or a simultaneous discrimination task, and it is often the case that differences in results are associated with these different paradigms (e.g., Boutet and Faubert, 2006 vs. Hildebrandt et al., 2010). Another point worth noting is that some studies have failed to include the critical inversion condition in their experiments. Indeed, the composite effect and the whole-part advantage are revealed via a significant interaction between the stimulus type and orientation (McKone et al., 2013). For example, the composite effect provides evidence that holistic information processing is unique to faces *because* the difference between recognition of composites and non-composites is reduced for inverted faces. Yet several studies have simply omitted to include an inverted condition, making it impossible to determine whether the expected interaction was present in older adults. The inclusion of a critical inversion condition is particularly important in this context because of the heterogeneity of function present in older adults. Indeed, conclusions that a given process is intact in older adults because age differences are not significant must be checked against the limitations of null findings, especially when variability is high. This problem can be partly avoided by testing for the presence of the effect itself (i.e., composite effect, whole-part advantage) in each age group because such effects are manifested via the significant finding of an interaction between stimulus type and orientation for each age group separately. Finally, much can be learned from simultaneously studying the functional and physiological processes underlying face recognition deficits in older adults. Future experiments adopting this approach should employ procedures that better match those that elicit deficits in older adults. Furthermore, all of the conditions necessary to replicate the effects that are the hallmark of face recognition should be included.

We end this discussion by arguing that more research is needed to decipher the relation, if any, of tasks that measure configural encoding with each other and with general face recognition abilities in both younger and older participants. Indeed, contradictory results across different tests of configural information processing exist not only for older adults but also in studies focusing on early development (reviewed by Taylor et al., 2004; Johnson, 2011). Some efforts at comparing different tests have already been undertaken (Konar et al., 2010; Richler et al., 2011; DeGutis et al., 2013), notably the study by Hildebrandt et al. (2010) which focuses on individual differences in the behavioral performance of young, middle-aged, and older adults on a total of twelve face recognition tasks. Despite the difficulties inherent in testing such large numbers of participants on so many tasks, we believe that such studies should be replicated given that testing the same participants on several tests of face recognition eliminates the confound of participant variability in cognitive function. Moreover, studies focusing on individual differences give researchers the opportunity to examine whether common processes are recruited during different tests of configural information processing. Studies adopting this approach with older participants should include a condition that will replicate the high false alarm rate that is the signature of face recognition deficits in this population.

Both perceptual deterioration and impaired configural processing map onto the first stages of face recognition when a representation is extracted from a perceived face. The presence of one or both deficits might result in erroneous feelings of resemblance between perceived and stored representations. The decision regarding whether or not the face is actually familiar would then depend on access to information regarding previous encounters with the face. The following hypothesis maps onto this latter stage.

## Decline in the Recollection of Contextual Information

Age-related deficits in face recognition have also been attributed to a decline in recollecting contextual or source information when perceived faces trigger a feeling of familiarity. This hypothesis emerged from the robust findings that face recognition deficits in older adults are characterized by higher false alarms to unfamiliar faces (for a review, see by Searcy et al., 1999). Within the context of the framework presented herein, a feeling of familiarity will arise when there is a match between a perceived representation and a stored representation. According to the contextual information hypothesis, additional information regarding the context under which the perceived information was previously encountered is necessary to correctly discriminate *seemingly* familiar from *truly* familiar faces. Therefore, correctly rejecting a face that appears familiar because it happens to resemble a stored representation requires the extra step of context recollection. However, if access to contextual information is impaired, then face recognition will be based mainly on familiarity judgments, leading to the high false alarm rates observed in older adults.

The four following studies have included manipulations aimed at testing the context recollection hypothesis directly, and all four support the notion that older adults are more likely than to younger adults to base their recognition decision on familiarity. First, Bartlett et al. (1991) reported that older adults produced more false positives than younger adults when judging whether faces are those of celebrities or novel unknown faces. Most importantly, the age difference was particularly pronounced for faces that were likely to yield feelings of familiarity because they had previously been presented in the context of the experiment. Second, Bartlett and Fulton (1991) showed that perceived familiarity of new faces is significantly correlated with incorrectly stating that the face had previously been presented in older adults. Third, Searcy et al. (1999) demonstrated that older adults do not show higher false alarms to conjunction faces constructed from the inner and outer features of two different faces while still showing higher false alarms to non-manipulated faces. They reasoned that because the perceptual information in conjunction faces poorly matches representations stored in memory, conjunctions should not seem familiar and are therefore easily rejected by older adults without the need to rely on context recollection (see also Rhodes et al., 2008). Finally, Edmonds et al. (2012) have specifically manipulated the familiarity and context of faces by presenting faces as lures in a session where participants were asked to judge personality traits, followed by the presentation of study faces where participants were asked to remember the faces for a memory test. The memory test included both the studied faces and the familiarized lures. While older adults displayed similar hit rates for studied faces and correction rejection for new foils, their ability to correctly reject familiarized lures was significantly impaired in comparison to the younger participants. These results support the contention that older adults rely more heavily on familiarity when making yes/no decisions in face recognition tasks.

Studies on the bystander effect, whereby bystanders are often mistakenly identified as perpetrators, are also relevant to the context recollection hypothesis (Searcy et al., 1999). This effect is thought to arise because the face of the bystander is perceived as familiar, and therefore context recollection is essential to correctly reject his/her face during lineup identification procedures. If the context recollection hypothesis is correct, older adults should be more likely to incorrectly identify the bystander as the perpetrator because they will base their judgment on perceived familiarity without recollecting information regarding the context in which the face was encountered. Indeed, a number of studies have demonstrated that older adults are more prone to the bystander effect than younger adults (reviewed by Memon et al., 2002). However, Searcy et al. (2001) failed to find higher false alarms to bystanders in older adults and Memon et al. (2002) failed to demonstrate a positive impact of context reinstatement on the bystander effect in older adults. This evidence suggests that the link between the bystander effect and difficulties in recollecting contextual information is equivocal.

One possible avenue of future research is to determine whether problems with context recollection are face-specific. On the one hand, there is evidence that older adults display inflated false alarms for other types of stimuli (reviewed by Searcy et al., 1999), such as semantic stimuli (e.g., Dywan and Jacoby, 1990; Ozen et al., 2010). On the other hand, familiarity-based responding is much more likely to yield to practical difficulties in older adults for faces than for other stimuli because (i) faces are the only category for which correct individual recognition is frequently crucial during social interactions and (ii) there is an accrual of memorized faces with increasing age (Chaby and Narme, 2009). Unfortunately, to our knowledge, no study has measured age differences on both faces and other objects using comparable yes/no recognition tasks.

While we did not distinguish between familiar and unfamiliar faces in the organizing framework, there is some evidence to suggest that they trigger different forms of processing (e.g., Bruce and Young, 1986; Johnston and Edmonds, 2009). In their review on the topic, Johnston and Edmonds (2009) suggest that processing of familiar and unfamiliar faces might differ early in the processing stream because with an unfamiliar face, “*we are unable to know which characteristics or image properties will be key to representing the identity of an individual*” (Johnston and Edmonds, 2009, p. 591). For example, an observer might focus on distinctive features as a strategy to later remember an unfamiliar face. In contrast, a face triggering feelings of familiarity might be carefully analyzed to identify remembered aspects of the face (Bruce and Young, 1986). With some creativity, researchers might devise ways to examine the treatment of unfamiliar faces by older adults not only during context recollection but in earlier stages as well. On a related note, it will be important for future investigations to distinguish between the results of laboratory experiments, where the distinction between familiar and unfamiliar stimuli is induced artificially, and the real-life task of face recognition where the context is likely to be more elaborate.

We also suggest that future studies take advantage of modern image manipulation techniques to investigate recognition confusions in older adults. For example, instead of using conjunction faces like those employed in Searcy et al. (1999), future studies could use morphing techniques to systematically vary the resemblance of new faces and old faces encountered in different contexts. One recent study has used this approach to demonstrate that older adults have more difficulty than younger adults in discriminating morphed faces (Lee et al., 2014; see also Hildebrandt et al., 2011). It would be interesting for follow-up studies to employ these techniques in a recognition task to test whether faces with greater similarity yield more false alarms in older adults. It might also be possible to combine morphing techniques with experimental manipulations borrowed from studies on source memory (e.g., Brown et al., 1995; Skinner and Fernandes, 2009; Rahhal et al., 2002; Luo et al., 2007) to examine if manipulations of the context under which a face was viewed has a differential impact on younger and older adults. Finally, because face recognition deficits in older adults have deleterious implications for eyewitness testimonies, further investigations into techniques that could improve recollecting contextual information via cues to source memories are worth pursuing.

## Conclusion

In this review, we surveyed evidence pertinent to our understanding of the processes implicated in age-related face recognition deficits. Our discussion began with the premise that this decline is special because it is not merely a manifestation of general impairments associated with aging (Hildebrandt et al., 2011) and because it does not generalize to recognition of other complex objects (Grady et al., 1994, Experiment I; Boutet and Faubert, 2006; Chaby et al., 2011; Meinhardt-Injac et al., 2014). We then presented a generic framework that served to organize our subsequent review of three hypotheses that have been proposed to explain the face recognition deficits seen in older adults.

The impaired sensory processing hypothesis states that older adults have difficulty recognizing faces because of impairments in low-level perceptual capacities such as acuity or contrast sensitivity. While several studies have established a link between processing of basic spatial frequency information and face recognition abilities in both younger and older adults, some of the evidence reviewed was equivocal. Suggested avenues for future research include mimicking age-related perceptual loss in younger adults, ascertaining the range of spatial frequencies critical for face processing in older adults, and distinguishing between perceptual deficits that produce idiosyncratic *versus* generalized impairments.

While perceptual deterioration is likely to contribute to age-related deficits in face recognition as well as in other cognitive abilities (e.g., Schneider and Pichora-Fuller, 2000), some have suggested that impairments in mechanisms specialized for face recognition must also be at play. The impaired configural processing hypothesis states that older adults have difficulties recognizing faces because of a deficit in encoding holistic and/or second-order information, both having been implicated in face recognition. This hypothesis provides the best match between the suggested vulnerability of face recognition in aging and the special processing style that underlies face recognition. Although the configural information hypothesis has received widespread attention in the literature in the past 10 years from both behavioral and imaging studies, the variety of procedures used and the contradictory effects reported, even sometimes in the same study (e.g., Boutet and Faubert, 2006; Hildebrandt et al., 2010), makes it difficult to judge its validity. Nonetheless, it is clear that older adults can, under certain circumstances, encode configural information, suggesting that a failure to encode this type of information is unlikely to be the principal determinant of recognition impairments. Future research in this direction should recruit larger sample sizes to take into account the heterogeneity inherent to the cognitive changes that occur with aging and include the necessary controls to eliminate alternative explanations unrelated to the special processes triggered by faces. Investigations into the conditions under which older adults can, and cannot, extract configural information from faces are also warranted.

The context recollection hypothesis was born out of the finding that older adults exhibit inflated false alarm rates to unfamiliar faces. It maps onto the final stages of the face recognition stream by stating that older adults are more likely to base their recognition decision on perceived familiarity because of difficulties in recollecting contextual information. Research conducted within this framework is promising because it provides an opportunity to translate laboratory findings into the real world situation of eyewitness testimonies. The evidence reviewed partially supports the context recollection hypothesis, suggesting that older adults recognize faces on the basis of perceived familiarity. Nonetheless, additional research using more modern image manipulation techniques (i.e., morphed images) as well as context reinstatement paradigms are needed to further establish its validity. Future studies should also distinguish between familiar and unfamiliar faces when comparing younger to older adults.

These three explanations are not mutually exclusive and all three may work in concert to result in the observed decline in face recognition in normal aging. Impaired contrast sensitivity, alongside diminished encoding of configural information (in certain conditions), would result in confusion when comparing perceived faces to stored representations in the first step of the face recognition process. As a result of this confusion, new faces are more likely to erroneously resemble stored representations and generate feelings of familiarity, especially because of the large number of faces that have been memorized by older adults (Chaby et al., 2001). Impaired access to contextual information would then prevent older adults from correctly rejecting new faces, leading to the high false alarm rate that is the signature of face recognition deficits in this population.

While the current review focuses on the functional aspects of face recognition deficits in older adults, past research points to a variety of plausible underlying physiological mechanisms. For example, reduced synaptic density (e.g., Hedden and Gabrieli, 2004; Kaup et al., 2011) could lead to reduced activation in low-level areas specialized in coding basic attributes such as contrast sensitivity, as well as in the areas of the face network specialized in processing configural information (Zhang et al., 2012). Deterioration of the hippocampus and the concomitant decline in episodic memory (Dickerson and Eichenbaum, 2010) would impede access to contextual information. These alterations could in turn lead to compensatory mechanisms such as activation in the prefrontal cortex, frontal cortex, and other associative areas (e.g., Reuter-Lorenz and Park, 2010). Finally, decreased efficiency in the different structures implicated in the face recognition process might also lead to *dedifferentiation* in the pattern of activation produced by faces in older adults (e.g., Grady et al., 1994; Lindenberger and Baltes, 1997; Park et al., 2004; Payer et al., 2006). More specifically, the brains of older adults may display diminished activation of the network of areas preferentially activated by faces and/or increased activation of areas implicated in more generic cognitive processes.

We have not attempted an exhaustive review of the literature on face recognition deficits in older adults in this paper and several explanations were omitted, either because they serve best to characterize the nature of the deficit (e.g., reduction in speed of processing: Salthouse, 1996; Pfutze et al., 2002; Rousselet et al., 2010; Rhodes and Anastasi, 2012; own-age bias: Fulton and Bartlett, 1991; Wiese et al., 2012; Verdichevski and Steeves, 2013) or because the proposed mechanisms are actually linked to one of the three hypotheses examined herein (e.g., processing of horizontal information: Narme et al., 2011; Obermeyer et al., 2012; context congruency: Meinhardt-Injac et al., 2014; changes in eye movements: Firestone et al., 2007; Chan et al., 2011). That is not to say that these other accounts should be ignored and in fact, we feel, like others, that the search for a single explanatory cause in aging studies is not likely to be fruitful. Instead, different techniques should be used to cast a broad net of investigation into several possible mechanisms that can then be eliminated or refined in light of the accumulated evidence. We hope that our review of three seemingly disparate and yet promising hypotheses illustrates the promise of this approach for our understanding of both aging and face recognition.

### Conflict of Interest Statement

The authors declare that the research was conducted in the absence of any commercial or financial relationships that could be construed as a potential conflict of interest.

## Footnotes

1 There is considerable debate regarding whether a dedicated system exists for faces, or whether faces are simply an example of expert object recognition, and we direct the reader to other sources for more details on this issue (see, e.g., Gauthier et al., 2003; Wallis, 2013, for alternative views).
